# Involvement of Melatonin, Oxidative Stress, and Inflammation in the Protective Mechanism of the Carotid Artery over the Torpor–Arousal Cycle of Ground Squirrels

**DOI:** 10.3390/ijms252312888

**Published:** 2024-11-29

**Authors:** Ziwei Hao, Yuting Han, Qi Zhao, Minghui Zhu, Xiaoxuan Liu, Yingyu Yang, Ning An, Dinglin He, Etienne Lefai, Kenneth B. Storey, Hui Chang, Manjiang Xie

**Affiliations:** 1 Department of Aerospace Physiology, Air Force Medical University, Xi’an 710032, China; haoziwei@stumail.nwu.edu.cn; 2 Shaanxi Key Laboratory for Animal Conservation, Northwest University, Xi’an 710069, China; hanyuting@stumail.nwu.edu.cn (Y.H.); 18109208398@163.com (Q.Z.); 202121731@stumail.nwu.edu.cn (M.Z.); liuxiaoxuan2@stumail.nwu.edu.cn (X.L.); yangyingyu@stumail.nwu.edu.cn (Y.Y.); anning@stumail.nwu.edu.cn (N.A.); 2021113086@stumail.nwu.edu.cn (D.H.); 3 INRAE, Unité de Nutrition Humaine, Université Clermont Auvergne, UMR 1019, F-63000 Clermont-Ferrand, France; entienne.lefai@inrae.fr; 4 Department of Biology, Carleton University, Ottawa, ON K1S 5B6, Canada; kenstorey@cunet.carleton.ca

**Keywords:** carotid artery, Daurian ground squirrels, melatonin, oxidative stress, inflammatory responses

## Abstract

Hibernating mammals experience severe hemodynamic changes over the torpor–arousal cycle, with oxygen consumption reaching peaks during the early stage of torpor to re-enter arousal. Melatonin (MT) can improve mitochondrial function and reduce oxidative stress and inflammation. However, the regulatory mechanisms of MT action on the vascular protective function of hibernators are still unclear. Morphology, hemodynamic, mitochondrial oxidative stress, and inflammatory factors of the carotid artery were assessed in ground squirrels who were sampled during summer active (SA), late torpor (LT), and interbout arousal (IBA) conditions. Changes were assessed by methods including hematoxylin and eosin staining, color Doppler ultrasound, ELISA, Western blots, and qPCR. Changes in arterial blood and serum melatonin were also measured by blood gas analyzer and ELISA, whereas mitochondrial oxidative stress and inflammation factors of primary vascular smooth muscle cells (VSMCs) were assessed by qPCR. (1) Intima-media carotid thickness, peak systolic velocity (PSV), end diastolic blood flow velocity (EDV), maximal blood flow rate (Vmax) and pulsatility index (PI) were significantly decreased in the LT group as compared with the SA group, whereas there were no difference between the SA and IBA groups. (2) PO_2_, oxygen saturation, hematocrit and PCO_2_ in the arterial blood were significantly increased, and pH was significantly decreased in the LT group as compared with the SA and IBA groups. (3) The serum melatonin concentration was significantly increased in the LT group as compared with the SA and IBA groups. (4) MT treatment significantly reduced the elevated levels of LONP1, NF-κB, NLRP3 and IL-6 mRNA expression of VSMCs under hypoxic conditions. (5) Protein expression of HSP60 and LONP1 in the carotid artery were significantly reduced in the LT and IBA groups as compared with the SA group. (6) The proinflammatory factors IL-1β, IL-6, and TNF-α were reduced in the carotid artery of the LT group as compared with the SA and IBA groups. The carotid artery experiences no oxidative stress or inflammatory response during the torpor–arousal cycle. In addition, melatonin accumulates during torpor and alleviates oxidative stress and inflammatory responses caused by hypoxia in vitro in VSMCs from ground squirrels.

## 1. Introduction

Hibernation is a survival strategy utilized by many mammals that includes significant physiological, morphological, and behavioral changes as well as an active reduction of metabolic rate in order for animals to survive the food scarcity and harsh natural environments of winter [1,2,3]. The major seasonal characteristic is a prolonged state of torpor [2]. Some small rodents, such as ground squirrels, undergo repeated and spontaneous interbout arousals (IBAs) during hibernation, where their body temperature rises to euthermic values (~37 °C) for about 24 h and then falls again as the animals re-enter torpor [4,5]. Hibernators also experience adaptive changes in vascular function while in torpor; for example, depression of endothelium-dependent vasodilator responses to acetylcholine and ATP occur in the carotid artery of hibernating golden hamsters (*Mesocricetus auratus*) [6], which are reversed during IBA, without damaging organ function [7]. Our previous study on Daurian ground squirrels (*Spermophilus dauricus*) showed that, in the IBA group, a decrease in reactive oxygen species (ROS) and pro-inflammatory factors, an increase in superoxide dismutase and anti-inflammatory factors, and in the torpor group, a restoration of endothelial permeability numbness led to controlled phenotypic changes of smooth muscle cells of the thoracic aorta [4]. In addition, it has been reported that the increased contractility of aortic vascular tissue during torpor returns to normal within 1.5 h after arousal and is associated with increased basal nitric oxide (NO) synthesis in European ground squirrels (*Spermophilus citellus*) [8].

The transition from torpor (low oxygen metabolism) to arousal (normal metabolism) is a high oxygen consumption process in Arctic ground squirrels (*Spermophilus parryii*) and thirteen-lined ground squirrels (*Spermophilus tridecemlinatus*) [9,10]. The rapid increase in metabolic rate during arousal may cause overproduction of ROS as compared with torpor in the plasma of Arctic ground squirrels [11,12]. Research has shown that oxygen consumption reached a peak during the second hour of the torpor to arousal transition. Furthermore, an increase in urate production in plasma catalyzed by elevated activity of xanthine oxidase indicated an induction of ROS production and hypoxia during this period in Arctic ground squirrels [11]. Hypoxia can trigger an increase in heart rate and peripheral arterial blood pressure but decreases stroke volume in non-hibernators [13]. Furthermore, hypoxia can lead to a decrease in arterial oxygen partial pressure (PO_2_), oxygen saturation, arterial blood partial pressure of carbon dioxide (PCO_2_) and an increase in arterial pH in humans [14,15,16]. Furthermore, the thickness of the thoracic aortic media increased after 45 min of hypoxia and ischemia in rats [17]. The expression of heat shock proteins (such as HSP60, a mitochondrial chaperone protein that regulates apoptosis-related proteins and inhibits apoptosis) is upregulated in pulmonary artery smooth muscle cells under hypoxic (3% O_2_) conditions [18]. Also, the hypoxia-inducible factor-1α (HIF-1α) can bind to hypoxia responsive elements in the promoter of the mitochondrial Lon protease gene (LONP1), preventing proteotoxicity during environmental and cellular stress and leading to upregulation of LONP1 expression under hypoxic conditions in humans [19]. Serum IL-1β, IL-6, IL-10 and TNF-α levels are elevated during hypoxia when healthy humans ascend to 3800 m [20]. In hibernators, the cerebral arterial oxygen partial pressure (PO_2_) and oxygen saturation are significantly decreased, with the hypoxia-associated protein HIF-1α accumulating in the brain of the IBA group compared to the torpor group and providing evidence for hypoxia in thirteen-lined ground squirrels [21]. Similarly, HIF-1α was reduced in brain tissue (which is supplied by the internal carotid artery) during torpor as compared with room temperature conditions among bats (*Hipposideros terasensis*) [22]. Along with a periodic decrease in O_2_ requirements during hibernation, blood O_2_ affinity increased due to a decrease in body temperature and the concentration of 2,3-diphosphoglycerol in red blood cells. This caused a decrease in O_2_ unloading to tissues, thereby contributing to limiting tissue oxidative stress [23,24,25]. Therefore, blood vessels exhibit a protective action for hypoxia in hibernators, making them a natural model for avoiding hypoxic damage.

Although the central mechanisms that control cyclic bouts of hibernation in ground squirrels are not clearly understood, there is evidence suggesting that melatonin (MT) is involved [26]. MT secreted by the pineal gland of the brain can synchronize endogenous signals of the circadian rhythm, allowing animals to adapt to rapid changes in rhythmic environmental conditions [27,28]. MT has a vasodilatory effect and can improve endothelial function through its free radical scavenging and antioxidant properties [29,30]. Moreover, MT can bind with receptors that are located in arteries, thereby protecting physiological functions under hypoxic conditions in non-hibernators [31]. For example, MT has a vasodilative effect on cerebral blood vessels of newborns and reduces cerebral inflammation after hypoxia in humans [32,33]. The antioxidant effect of MT can alleviate the inhibitory effect on NO-mediated vascular relaxation by chronic hypoxia in the ovine common carotid artery [32,34]. Daily MT treatment protected both the structural and functional integrity of the aortic endothelium in mice and human umbilical vein endothelial cells (i.e., a form of ECs) against oxidative stress and ischemia-induced damage [35]. In hibernators, MT levels increase during torpor in the plasma of golden-mantled ground squirrels (*Spermophilus lateralis*) [36]. Furthermore, production of MT can help brain mitochondria to work more efficiently during periods of extreme energy needs such as during arousal from torpor in thirteen-lined ground squirrels [37]. Increased levels of MT can also inhibit the effects of angiotensin II in the cardiovascular system during torpor in thirteen-lined ground squirrels [38].

The protective effect of MT on blood vessels under hypoxic conditions in hibernators is currently unclear. Thus, we hypothesize that increased secretion of melatonin during torpor plays a protective role in dealing with oxidative stress and inflammation in the carotid arteries of ground squirrels during the “torpor–arousal” cycle. In the present study, serum MT concentration was measured, and carotid artery tissue was examined via hematoxylin and eosin (HE) staining, color Doppler ultrasonography, and arterial blood gas analysis. Oxidative stress and inflammatory related factors were detected in ground squirrels from summer active (SA), late torpor (LT), and interbout arousal (IBA) conditions. Furthermore, vascular smooth muscle cells (VSMCs) from the carotid artery were separated and cultured under 6% hypoxia conditions and were used as an in vitro model of hypoxia to test the protective effects of MT and evaluate oxidative stress and inflammatory related factors.

## 2. Results

### 2.1. Changes in Carotid Artery Intima-Media Thickness of Ground Squirrels

**Figure 1:**
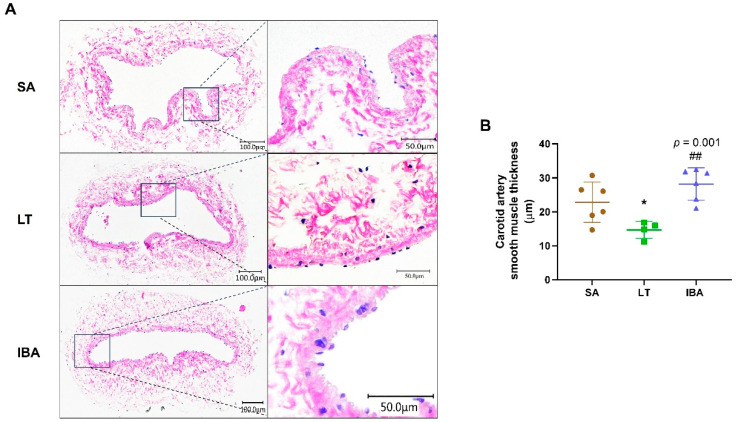
HE staining results for carotid arteries in ground squirrels. (**A**) Typical images of HE staining of carotid arteries at three different groups. Tissue scales in the left column are 100 µm and in right column are 50 µm, (**B**) intima-medial measure thickness at three random locations on each carotid artery. SA: summer active, LT: late torpor, IBA: interbout arousal. *n* = 4~6. Data are mean ± SD. Statistically significant differences are denoted as follows: * *p* < 0.05, as compared with the SA group and ## *p* < 0.01, as compared with the LT group.

The thickness of the intima-media of the carotid artery was significantly reduced by 35.5% (*p* < 0.05) in the LT group as compared with the SA group. By contrast, the intima-media thickness was dramatically improved by 91.8% (*p* = 0.001) in the IBA group compared to the LT group (Figure 1).

### 2.2. Changes in Carotid Artery Hemodynamics of Ground Squirrels

**Figure 2:**
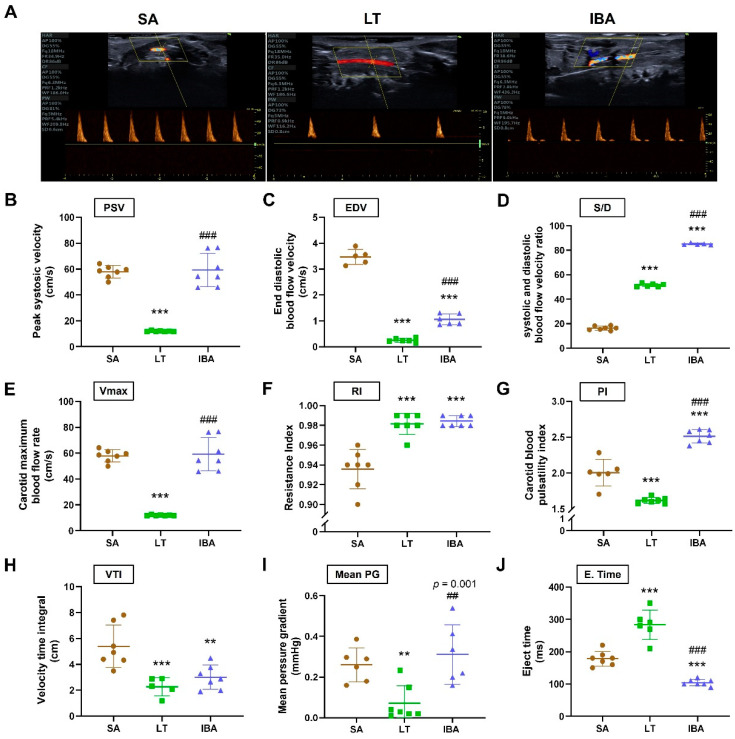
Hemodynamics of carotid arteries in ground squirrels. (**A**) Typical images of carotid artery hemodynamics at three different groups, (**B**) peak systolic velocity (PSV, cm/s), (**C**) end diastolic blood flow velocity (EDV, cm/s), (**D**) systolic and diastolic blood flow velocity ratio (S/D), (**E**) maximum carotid blood flow rate (Vmax, cm/s), (**F**) resistance index (RI), (**G**) carotid arteries pulsatility index (PI), (**H**) velocity time integral (VTI, cm), (**I**) mean pressure gradient (mean PG, mmHg), (**J**) ejection time (E. time, ms). SA: summer active, LT: late torpor, IBA: interbout arousal. *n* = 6~7. Data are mean ± SD. Statistically significant differences are denoted as follows: ** *p* < 0.01, *** *p* < 0.05, as compared with the SA group, and ## *p* < 0.01, ### *p* < 0.001, as compared with the LT group.

Compared with the SA group, the peak systolic velocity (PSV), end diastolic blood flow velocity (EDV) and maximal blood flow rate (Vmax) of the carotid arteries were significantly decreased by 79.4% (*p* < 0.001), 93.1% (*p* < 0.001) and 79.4% (*p* < 0.001), respectively, in the LT group. Besides, EDV showed a significantly decrease by 70.6% (*p* < 0.001) in the IBA group compared to the SA group. PSV, EDV and Vmax were significantly increased in the IBA group by 456.9% (*p* < 0.001), 325.7% (*p* < 0.001) and 397.6% (*p* < 0.001), respectively, as compared with the LT group (Figure 2B,C,E). In addition, the systolic and diastolic blood flow velocity ratio (S/D) was markedly improved by 215% (*p* < 0.001) and 420.5% (*p* < 0.001), respectively, in the LT and IBA groups compared to the SA group; meanwhile, it was dramatically improved by 65.2% (*p* < 0.001) in the IBA group as compared with the LT group (Figure 2D).

The carotid resistance index (RI) of the carotid arteries was significantly increased by 4.9% (*p* < 0.001) in the LT group and 5.2% (*p* < 0.001) in the IBA group compared to the SA group, but there was, no significant difference between the LT and IBA groups (Figure 2F). The pulsatility index (PI) of the carotid arteries was markedly decreased by 19.3% (*p* < 0.001) in the LT group and significantly increased by 25.2% (*p* < 0.001) in the IBA group compared to the SA group and was significantly increased by 55.2% (*p* < 0.001) in the IBA group as compared with the LT group (Figure 2G).

The velocity time integral (VTI) and mean pressure gradient (mean PG) of the carotid arteries were dramatically reduced by 58.1% (*p* < 0.001) and 72.4% (*p* < 0.01), respectively, in the LT group; also, VTI was significantly decreased by 44.5% (*p* < 0.01) in the IBA group as compared with the SA group. In addition, the mean PG was significantly increased by 333.5% (*p* < 0.001) in the IBA group as compared with the LT group (Figure 2H,I). The eject time (E. Time) of the carotid arteries was significantly elevated by 58.7% (*p* < 0.001) in the LT group and significantly decreased by 41.6% (*p* < 0.001) in the IBA group as compared with SA, and it was significantly reduced by 63.2% (*p* < 0.001) in the IBA compared to the LT group (Figure 2J).

### 2.3. Arterial Blood Gas Value of Ground Squirrels

**Figure 3:**
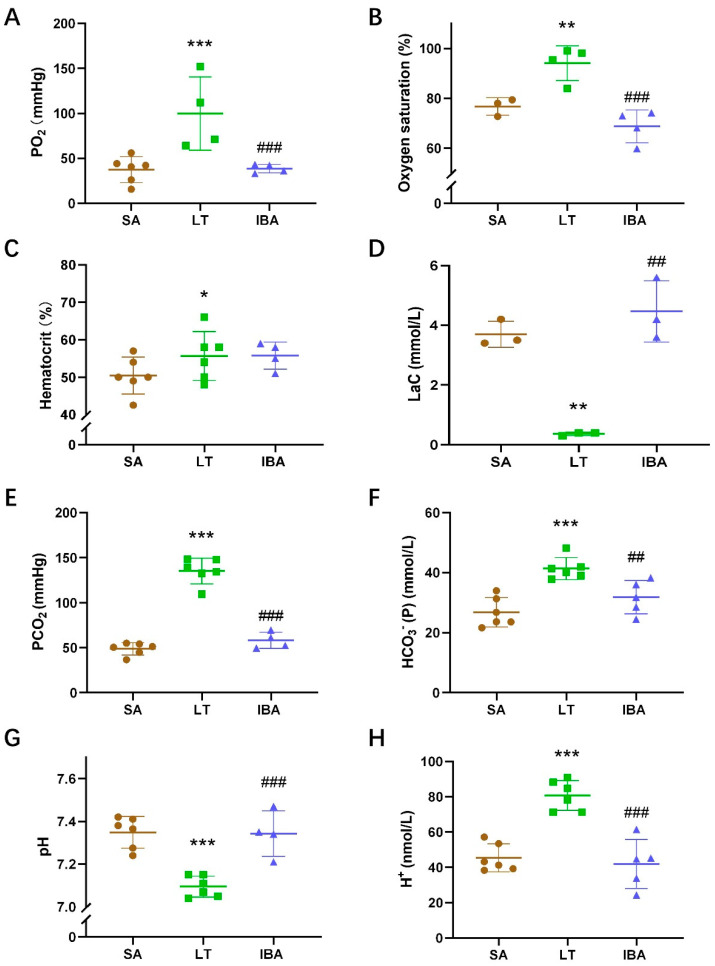
Levels of PO_2_, oxygen saturation, hematocrit, lactic acid, pH, H^+^, PCO_2_ and HCO_3_^−^ in arterial blood of ground squirrels. (**A**) Arterial blood oxygen partial pressure (PO_2_), (**B**) arterial blood oxygen saturation, (**C**) arterial blood hematocrit, (**D**) arterial blood lactic acid (LaC), (**E**) arterial blood partial pressure of carbon dioxide (PCO_2_), (**F**) arterial blood HCO_3_^−^, (**G**) arterial blood pH, (**H**) arterial blood H^+^. SA: summer active, LT: late torpor, IBA: interbout arousal, *n* = 3~6. Data are mean ± SD. Statistically significant differences are denoted as follows: * *p* < 0.05, ** *p* < 0.01, *** *p* < 0.001, as compared with the SA group, and ## *p* < 0.01, ### *p* < 0.001, as compared with the LT group.

Compared with the SA group, the partial pressure of oxygen (PO_2_), oxygen saturation, and hematocrit of arterial blood were significantly increased by 183% (*p* < 0.001), 18% (*p* < 0.01) and 10.4% (*p* < 0.05), respectively, in the LT group. In addition, PO_2_ and oxygen saturation of the arterial blood were dramatically reduced by 63.6% (*p* < 0.01) and 23% (*p* < 0.001), respectively, in the IBA group compared to the LT group (Figure 3A–C). The lactate of the arterial blood was significantly decreased by 90.1% (*p* < 0.01) in the LT group compared to the SA group, and it was markedly elevated by 927.3% (*p* < 0.01) in the IBA group compared to the LT group (Figure 3D).

The partial pressures of carbon dioxide (PCO_2_) and [HCO_3_^−^] in arterial blood were dramatically elevated by 177.1% (*p* < 0.001) and 54.1% (*p* < 0.001), respectively, in the LT group compared to the SA group. However, they were dramatically reduced by 56.9% (*p* < 0.001) and 23% (*p* < 0.01), respectively, in the IBA group as compared with the LT group (Figure 3E,F).

The arterial blood pH was significantly decreased in the LT group as compared with the SA group but was significantly increased in the IBA group as compared with the LT group (Figure 3G). The H^+^ level of the arterial blood was dramatically elevated by 77.7% (*p* < 0.001) in the LT group as compared with the SA group, but then decreased significantly by 48% (*p* < 0.001) in the IBA group compared to the LT group (Figure 3H).

### 2.4. Melatonin Level in Serum of Ground Squirrels

**Figure 4:**
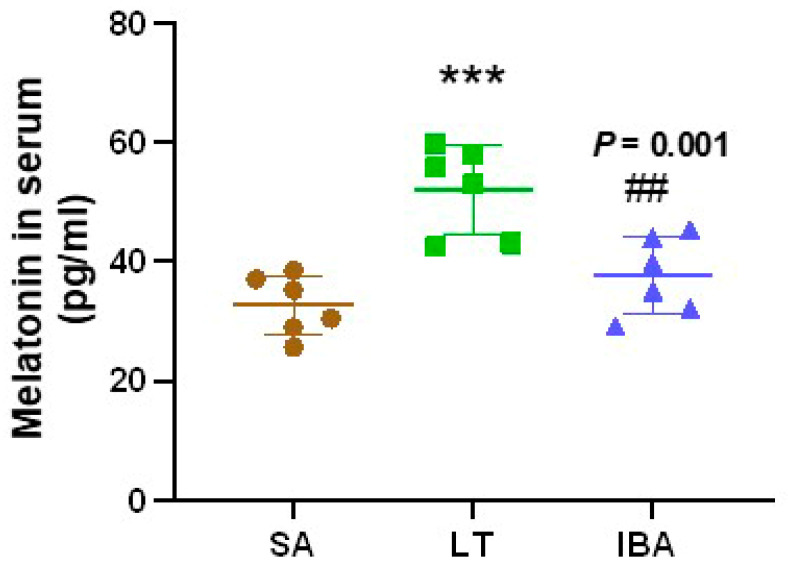
Determination of melatonin concentration in serum of ground squirrels by ELISA. SA: summer active, LT: late torpor, IBA: interbout arousal. *n* = 6. Data are mean ± SD. Statistically significant differences are denoted as follows: *** *p* < 0.001, as compared with the SA group, and ## *p* < 0.01, as compared with the LT group.

The melatonin concentration in the serum was significantly increased by 59.4% (*p* < 0.001) in the LT group as compared with the SA group, but it was significantly decreased by 27.7% (*p* = 0.001) in the IBA group as compared with the LT group (Figure 4).

### 2.5. The Effect of MT on Oxidative Stress and Inflammatory Factor Expression in Hypoxia-Induced Primary Cultured VSMCs of Ground Squirrels

**Figure 5:**
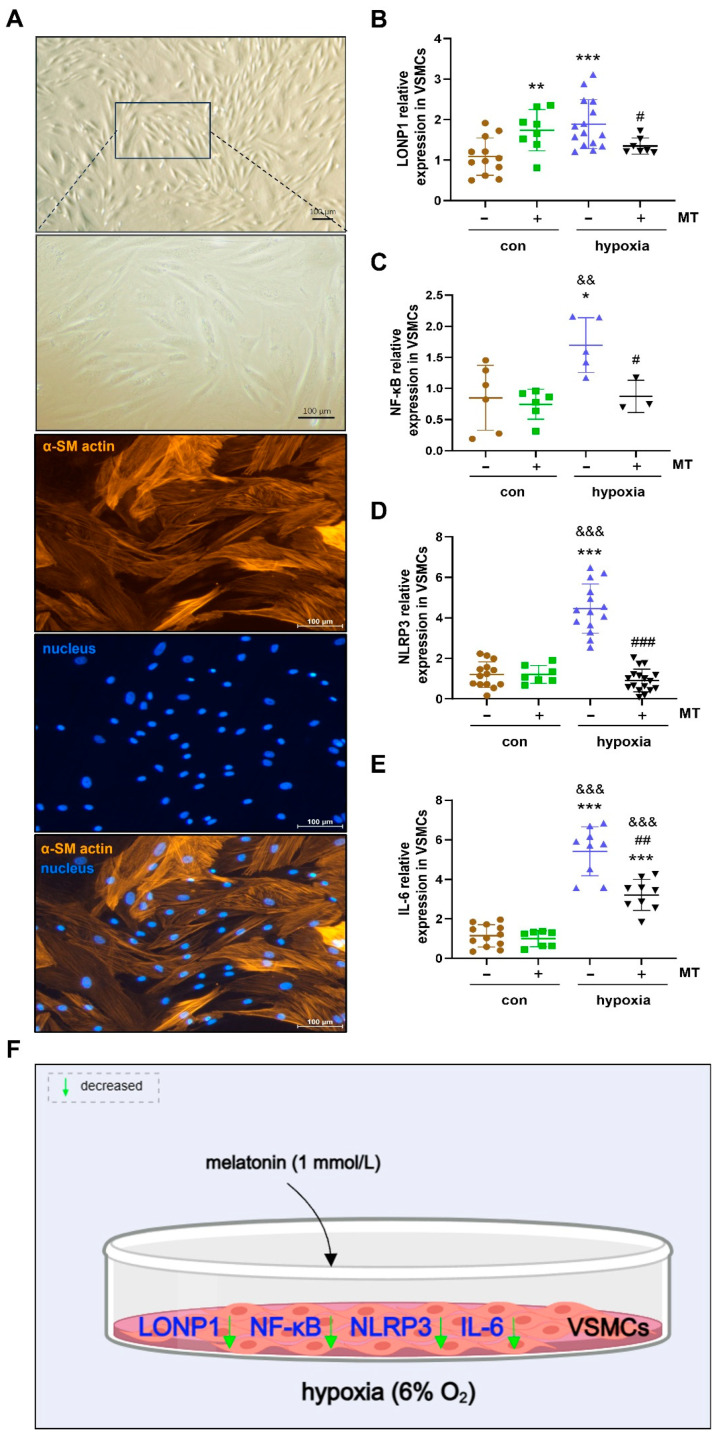
Carotid artery VSMC culture and characterization as well as the mitochondrial stress marker LONP1 and mRNA expression levels of inflammatory factors in ground squirrels. (**A**) Primary cultured smooth muscle cells and immunofluorescence was used to detect α-SM actin and nucleus (scale bar = 100 μm), (**B**) LONP1 mRNA expression level, (**C**) NF-κB mRNA expression level, (**D**) NLRP3 mRNA expression level, (**E**) IL-6 mRNA expression level, (**F**) summary of results for VSMCs. VSMCs: vascular smooth muscle cells, LONP1: Lon protease 1 mitochondrial, NF-κB: nuclear factor kappa-B, NLRP3: nucleotide-binding oligomerization domain-like receptor protein 3, IL-6: interleukin- 6. Data are mean ± SD. Statistically significant differences are denoted as follows: * *p* < 0.05, ** *p* < 0.01, *** *p* < 0.001, as compared with the control group, && *p* < 0.01, &&& *p* < 0.001, as compared with con + MT treated group, and # *p* < 0.05, ## *p* < 0.01, ### *p* < 0.001 as compared with hypoxia group.

The primary cultured VSMCs from the carotid artery were mainly in the shape of a long shuttle, a band, or a triangle. The nucleus was in the center of the cells, which were dense and showed a multilayered structure with the typical “peaks and valleys”. Specific α-SM actin immunofluorescence was used for actin staining identification of the VSMCs that were stained positive, having clear filaments radiating to the poles of the cell in the cytoplasm parallel to the long axis of the cell (Figure 5A).

Under hypoxia conditions, the LONP1 (an oxidative stress response protein) mRNA expression level was significantly elevated by 73.1% (*p* < 0.01) in VMSCs as compared with the control group. However, MT treatment (1 mmol/L) abrogated the hypoxia-induced increase of LONP1 mRNA expression in the VMSCs (Figure 5B,F). In addition, the mRNA expression levels of NF-κB, NLRP3 and IL-6 (markers of inflammation) were significantly increased by 171.8% (*p* = 0.01), 272.7% (*p* < 0.001) and 372.3% (*p* < 0.001), respectively, compared to the control group of VSMCs under hypoxia conditions. However, MT treatment abrogated the hypoxia-induced increase of NF-κB, NLRP3 and IL-6 mRNA expression in VMSCs (Figure 5C–F).

### 2.6. Oxidative Stress Levels in Carotid Arteries of Ground Squirrels

**Figure 6:**
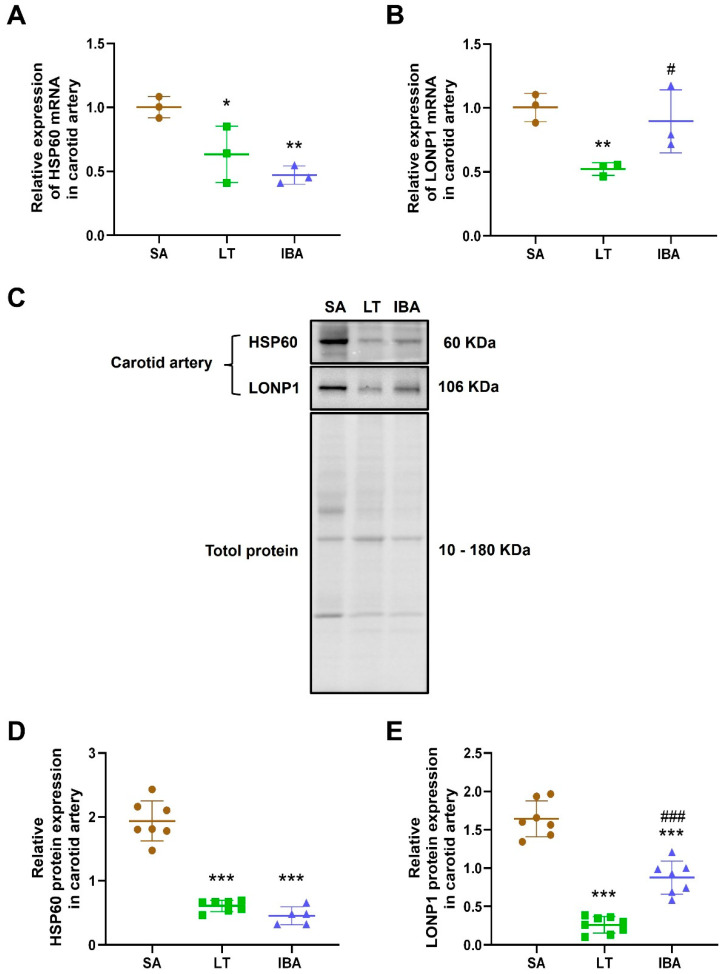
Expression levels of HSP60 and LONP1 mRNA and protein in carotid arteries of ground squirrels at three different groups. (**A**) Statistical graph of HSP60 mRNA expression levels in carotid arteries, (**B**) statistical graph of LONP1 mRNA expression levels in carotid arteries, (**C**) typical Western blot images of HSP60 and LONP1 proteins, (**D**) statistical graph of HSP60 protein expression levels in carotid arteries, (**E**) statistical graph of LONP1 protein expression levels in carotid arteries. SA: summer active, LT: late torpor, IBA: interbout arousal. HSP60: heat shock protein 60, LONP1: Lon protease 1 mitochondrial. *n* = 3~8. Data are mean ± SD. Statistically significant differences are denoted as follows: * *p* < 0.05, ** *p* < 0.01, *** *p* < 0.001, as compared with the SA group, and # *p* < 0.05, ### *p* < 0.001, as compared with the LT group.

The levels of HSP60 and LONP1 mRNA in the carotid arteries were significantly decreased by 36.8% (*p* < 0.05) and 48.05% (*p* < 0.01), respectively, in the LT group and the level of HSP60 mRNA was dramatically decreased by 53% (*p* < 0.01) in the IBA group as compared with the SA group. In addition, the level of LONP1 mRNA was significantly elevated by 10.96% (*p* < 0.05) in the IBA compared to the LT group (Figure 6A,B).

The expression of HSP60 and LONP1 protein in the carotid artery was significantly decreased by 63.8% (*p* < 0.001) and 83.6% (*p* < 0.001), respectively, in the LT group, and significantly reduced by 67% (*p* < 0.001) and 49.9% (*p* < 0.001), respectively, in the IBA group compared to the SA group. The LONP1 protein expression in the carotid artery was significantly elevated by 205.8% (*p* < 0.001) in the IBA group as compared with the LT group (Figure 6C–E).

### 2.7. Inflammatory Factor Levels in Carotid Arteries of Ground Squirrels

**Figure 7:**
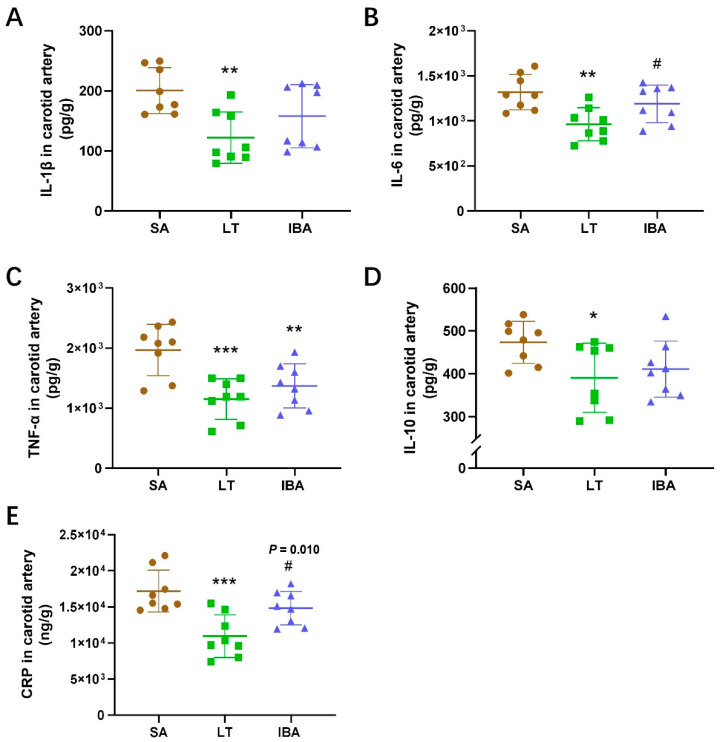
Levels of inflammatory factors in carotid arteries of ground squirrels as assessed by ELISA. (**A**) IL-1β concentration, (**B**) IL-6 concentration, (**C**) TNF-α concentration, (**D**) IL-10 concentration, (**E**) CRP concentration. SA: summer active, LT: late torpor, IBA: interbout arousal, IL-1β: interleukin-1β, IL-6: interleukin-6, TNF-α: tumor necrosis factor-α, IL-10: interleukin 10, CRP: C-reactive protein. *n* = 8. Data are mean ± SD. Statistically significant differences are denoted as follows: * *p* < 0.05, ** *p* < 0.01, *** *p* < 0.001, compared to the SA group, and # *p* < 0.05, compared to the LT group.

The pro-inflammatory factors IL-1β, IL-6, and TNF-α in the carotid artery were significantly reduced by 39% (*p* < 0.01), 26.9% (*p* < 0.01) and 41.4% (*p* < 0.001), respectively, in the LT group compared to the SA group. In addition, TNF-α in the carotid artery was significantly reduced by 30.4% (*p* < 0.01) in the IBA group as compared with the SA group, and IL-6 was significantly elevated by 23.4% (*p* < 0.05) in the IBA group as compared with the LT group (Figure 7A–C). CRP and the anti-inflammatory factor IL-10 in the carotid artery was dramatically reduced by 36.4% (*p* < 0.001) and 17.5% (*p* < 0.05), respectively, in the LT group as compared with the SA group. Furthermore, CRP in the carotid artery was dramatically increased by 35.6% (*p* = 0.010) in the IBA group compared to the LT group (Figure 7D,E).

## 3. Discussion

**Figure 8:**
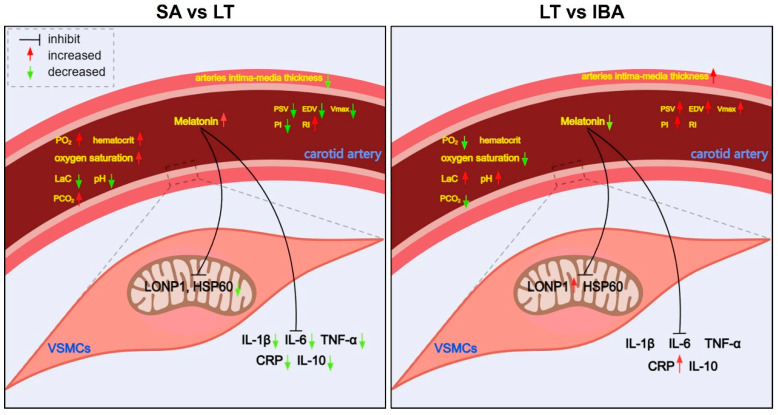
Diagrammatic representation of blood gas, melatonin, carotid artery function, mitochondrial stress and inflammation related to molecular protein expression in three groups of ground squirrels. The left image shows a comparison of SA vs. LT groups, and the right image shows LT vs. IBA groups. SA: summer active, LT: late torpor, IBA: interbout arousal, PO_2_: arterial blood oxygen partial pressure, PCO_2_: arterial blood partial pressure of carbon dioxide, LaC: arterial blood lactic acid, PSV: peak systolic velocity, EDV: end diastolic blood flow velocity, Vmax: maximum carotid blood flow rate, PI: carotid arteries perfusion index, RI: resistance index, VSMCs: vascular smooth muscle cells, LONP1: Lon protease 1 mitochondrial, HSP60: heat shock protein 60, IL-1β: interleukin-1β, IL-6: interleukin-6, TNF-α: tumor necrosis factor-α, IL-10: interleukin 10, CRP: C-reaction protein.

Blood vessels are composed of endothelial cells, smooth muscle cells and the basement membrane [39]. Vascular endothelial cells can regulate vascular tone, proliferation, and migration of VSMCs and inflammatory responses through secretion of vasoactive substances such as NO [40]. VSMCs, the main cells of the media vessel wall, can control blood flow by contracting or relaxing in response to external stimuli and play an important role in vascular pathologies [41]. In this study, the thickness of the intima-media of the carotid artery was reduced in the LT group compared to the SA group, whereas it was increased in the IBA as compared with the LT group (Figure 1 and Figure 8). Similarly, our previous research reported that the thickness of the intima-media of the thoracic aorta increased in the IBA group compared to the SA group, and the lumen was significantly narrowed in the thoracic aorta of the IBA group compared to the LT group in ground squirrels [4]. In addition, the mid-membrane of the aortic arch was also thickened and the number of VSMCs was significantly increased in torpid black bears (*Ursus americanus*) [42]. The varied timing of vessel wall thickening is possibly due to the specific species or tissues. Thickening of the pulmonary artery wall was also seen after 28 days of low-pressure hypoxia [43], and the proliferation ability of aortic VSMCs was enhanced when subjected to intermittent hypoxia in rats [44]. However, the present study indicated that there was no significant difference in carotid intima-media thickness between SA and IBA ground squirrels. Therefore, the recovery vessel wall thickness of the IBA group (equal to the SA group) may be due to increased metabolism, restored vascular function, or reduced lumen area in the IBA group.

Typical ground squirrel hibernation is characterized by prolonged periods of torpor with a significantly reduced heart rate, blood pressure, and blood flow that is interrupted every few weeks by brief interbout arousals (<24 h) during which blood flow fluctuates dramatically [45]. In the present study, the PSV, EDV, and Vmax of the carotid artery were significantly reduced in the LT group as compared with the SA group, whereas they were significantly increased in the IBA group as compared with the LT group (Figure 2B,C,E and Figure 8). In non-hibernators such as humans, ischemia and reperfusion injury are caused by the recovery of tissue blood flow after ischemic events [46]. However, our previous research reported that during torpor–arousal cycles, the thoracic aorta of ground squirrels can induce controlled phenotypic switching of VSMCs via changes in oxidative stress and inflammation levels caused by ischemia hypoxia, in order to resist ischemia–reperfusion injury [4].

The parameters RI and PI reflect blood flow resistance and depend on the degree of peripheral arterial wall stiffness or compliance [47]. Our data showed that the carotid RI was increased, whereas PI was decreased in the LT group as compared with the SA group in ground squirrels. RI and PI were also increased in the IBA group compared to the SA group, and PI was elevated in the IBA group compared to LT ground squirrels (Figure 2F,G and Figure 8). Consistent with the increase in carotid artery RI in young obese males [47], the changes in RI during hibernation suggest decreased carotid artery compliance and/or increased vascular resistance in hibernating ground squirrels, which may be due to excessive adipose tissue storage before hibernation. Furthermore, the reduced PI during torpor also depends on the volume blood flow and systemic circulatory factors including the reduced heart rate that is characteristic of torpor [47], as was reported in our previous study [48]. Moreover, PI = (systolic velocity − diastolic velocity)/systolic velocity [47], and systolic velocity increased more than diastolic velocity in the IBA group (Figure 2B,C and Figure 8), such that PI was significantly elevated in the IBA group compared to the LT group. In another study on Arctic ground squirrels focused on the re-entering arousal process, it was shown that pulmonary gas exchange did not keep up with oxygen demand, resulting in a decrease in hemoglobin saturation to roughly 60% [21,49]. Thus, in the early stage of transitioning from torpor to arousal, there must be a brief lack of oxygen supply due to the mismatch between accelerated blood circulation and respiratory rate.

Mammalian hibernators experience a decrease in body temperature, heart rate, ventilation, and metabolic rate during torpor, which means a concomitant downward adjustment of the oxygen consumption rate [49,50]. In hedgehogs (*Erinaceus europaeus*) and golden-mantled ground squirrels, the blood O_2_ affinity was significantly increased during torpor as compared with the SA group [51,52]. In the current study, the arterial blood PO_2_, hematocrit and oxygen saturation levels were significantly increased in the LT group, whereas they were decreased to the same level in the IBA group compared to the SA group (Figure 3A–C and Figure 8). This is consistent with the elevated carotid artery PO_2_ level found in Richardson’s ground squirrels (*Urocitellus richardsonii*) during hypothermia experiments, and the elevated hematocrit during torpor in brown bears (*Ursus arctos*) [25,50]. The increase in hematocrit is caused by a decrease in plasma/blood volume due to dehydration during torpor and not by an increased production of erythrocytes [53]. Besides, in the present study, arterial PO_2_ (105 mmHg) and oxygen saturation (94%) in the LT group were higher than that in the SA group (PO_2_ 42 mmHg and oxygen saturation 80%) of ground squirrels, almost close to that of non-hibernators. For example, systemic arterial oxygen saturation was about 95% and PO_2_ was 138 mmHg in healthy rats [54,55]. Consistently, the PO_2_ in torpor (99 mmHg) is higher than that in the SA group (65 mmHg) in thirteen-line ground squirrels [56]. Therefore, the oxygen reserve is increased in LT ground squirrels to provide oxygen when they re-enter arousal. On the contrary, the oxygen supply may become limited during arousal thermogenesis. For example, arterial PO_2_ falls to a minimum of 7 mmHg from the early stages of torpor to re-entering arousal (with the body temperature 10 °C) in Arctic ground squirrels [21]. In addition, the lactate concentration increases in brain tissue under peak oxygen consumption during arousal from torpor in bats, which suggests that animals experience oxygen deficiency during arousal reperfusion [57]. Moreover, consistent with the increased lactate concentration measured in the arterial blood of the IBA group as compared with the LT group in our study (Figure 3D and Figure 8), the lactate concentration also increased in the brain of non-hibernators under hypoxia [58]. However, in the present study, samples of the IBA group were taken from late-arousal ground squirrels (with a body temperature ~36 °C), and as the awakening time extended, the hypoxia state of the arousal from torpor was alleviated, so arterial PO_2_ and oxygen saturation in the IBA group were equal to those in the SA group.

CO_2_ solubility is increased in all body fluids of hibernators when body temperature decreases during torpor [49]. Similarly, in the present study, arterial blood PCO_2_ was significantly increased in the LT group as compared with the SA group, and there was no difference between the SA and IBA groups (Figure 3E and Figure 8). The decrease in ventilation leads to a significant increase in blood CO_2_, which results in respiratory acidosis (less CO_2_ removed than metabolically produced), as reported in torpid marmots (*Marmota jauiventris*), golden hamsters (*Mesocricetus aureus*) and dormice (*Glis glis*) [59,60,61]. Respiratory acidosis can induce initial metabolic suppression, either directly by reducing general body metabolism or indirectly by its depressant effect on central integrative processes in ground squirrels (*Citellus lateralis*) and European hamsters (*Cricetus cricetus*) [62,63]. In addition, arterial pH was significantly decreased and [HCO_3_^−^] was significantly increased in the LT group as compared with the SA group, but showed no difference between SA and IBA ground squirrels (Figure 3F,G and Figure 8), which is consistent with the decrease in blood pH from 7.45 to 7.42 and the increase of [HCO_3_^−^] from 30.3 to 37.4 mEq/L in golden mantled ground squirrels (*Spermophilus lateralis*) in torpor [60]. However, blood pH is unchanged during torpor as compared with the SA group in thirteen-lined ground squirrels [64]. Altogether, hibernators use pH-state regulation as a means of metabolic repression [60]. Moreover, consistent with our results (pH decreased and [HCO_3_^−^] increased in LT as compared with the SA group), hypercapnia and respiratory acidosis lower the threshold for shivering, inhibit cold-induced non-shivering thermogenesis and depress the metabolic rate in golden mantled ground squirrels, golden hamsters and dormice in order to maintain a hypometabolic and hypothermic state during torpor [61,65,66].

Melatonin can be found in all living organisms and exerts antioxidant effects via its expected long-term effects and being able to control cell and tissue function around the clock [67,68]. In our study, the melatonin concentration in serum was dramatically increased in the LT group compared to the SA group and showed no difference between SA and IBA ground squirrels (Figure 4 and Figure 8). Additionally, studies have shown that the MT receptor signal plays a protective role during the extreme physiological transition from torpor to arousal in thirteen-lined ground squirrels [37]. MT can also improve mitochondrial function, reduce oxidative stress, and increase respiratory chain activity [69,70]. LONP1, induced under oxidative stress and hypoxia conditions, is an essential mitochondrial protease that is crucial for maintaining mitochondrial protease homeostasis and reducing cellular stress, supporting cell viability in response to acute cellular stress [19,71]. In addition, NF-κB (Nuclear Factor κB) can be activated by oxidative stress [72], and it activates the expression of nucleotide-binding oligomerization domain-like receptor protein 3 (NLRP3), which can be inhibited by MT, thereby suppressing pro-inflammatory factor maturation and secretion [73,74]. In the current study, MT treatment abrogated the hypoxia-induced increase of LONP1, NF-κB, NLRP3 and IL-6 mRNA expression in VMSCs (Figure 5B–F), which is consistent with MT inhibition of the hyperactivity of NLRP3 inflammasomes by inhibiting NF-κB pathway activity under hypoxia in neonatal rats [75]. In addition, MT treatment reversed the increase in TNF, IL-6, and NF-κB expression under hypoxia/reoxygenation conditions in human primary villous cytotrophoblasts cells [76]. Therefore, our findings have indicated that MT can protect against oxidative stress and inflammation in VSMCs from ground squirrels under hypoxic conditions.

As previously mentioned, mitochondrial ROS increased during high oxygen consumption in the early stage of torpor to re-entering arousal, leading to oxidative stress [9,10]. Oxidative stress-mediated mitochondrial dysfunction stimulates the upregulation of mitochondrial HSP60 and ultimately initiates inflammatory pathways [77]. In the present study, the expression levels of HSP60 mRNA and protein were significantly reduced in the LT and IBA groups as compared with the SA group, and there was no difference between the LT and IBA groups (Figure 6A,C,D and Figure 8), which is consistent with the mRNA expression of HSP60, which showed no difference between the torpor and arousal groups in the hypothalamus of golden-mantled ground squirrels [78]. Additionally, protein expression of HSP60 increased in white adipose tissue during the transitory periods of the torpor–arousal cycle in thirteen-lined ground squirrels [79]. The different expression trends of HSP60 appear to be because white adipose tissue provides the fuel to support energy needs for ground squirrels to re-enter arousal, leading to a high metabolic rate and oxygen consumption. In the present study, the expression of LONP1 mRNA and protein in the carotid artery was decreased in the LT and IBA groups as compared with the SA group but increased in the IBA group as compared with LT ground squirrels (Figure 6B,C,E and Figure 8). The LONP1 expression level was highest in tissues with high energy demands such as heart, brain, liver, and skeletal muscles, acting to maintain mitochondrial function [71]. In addition, hypoxia upregulates LONP1, responding to oxidative stress by abrogating deleterious processes that threaten cell survival through its protease and chaperone activity [80]. Therefore, the hypoxia caused in the early stage of arousal from torpor, that, in turn, leads to oxidative stress, can be alleviated during the late-arousal period.

Oxidative stress can activate many transcription factors that promote the expression of inflammatory cytokines and anti-inflammatory molecules [72]. In non-hibernators, such as humans, increased levels of oxidative stress and inflammation in the carotid artery leads to tissue ischemia and is believed to impair cerebrovascular function [81,82]. In addition, NF-κB can trigger the activation of the NLRP3 inflammasome, and the activation of both the NLRP3 inflammasome and the NF-κB innate immune pathway results in the overproduction of pro-inflammatory factors such as IL-1β and TNF-α [73,81,83,84]. In the present study, the expression of pro-inflammatory factors TNF-α, IL-1β, IL-6, and CRP (a suggestive or discriminatory indicator of bacterial infection), and anti-inflammatory factor IL-10 in the carotid artery were significantly and consistently decreased in the LT group compared to the SA group (Figure 7 and Figure 8). Similarly, no inflammation was observed in the arteries of torpid brown bears [85]. However, a systemic inflammatory response was observed in torpid Arctic ground squirrels and brown bears, which is consistent with our previous study that showed that pro-inflammatory factors (TNF-α, IL-1β, IL-6, and CRP) were elevated and anti-inflammatory factors (IL-10 in the serum) were decreased in the LT group compared to the SA group [4,5,86,87]. Furthermore, in the current study, although IL-6 and CRP were significantly increased in the IBA group, as compared with the LT group, there was no difference in IL-1β, IL-6, CRP, or IL-10 between the SA and IBA groups, and TNF-α was significantly reduced in the IBA group as compared with the SA group (Figure 7 and Figure 8). Thus, the carotid arteries exhibit no inflammatory response and maintain their structure and function during the torpor–arousal cycle despite rapid changes in blood flow velocity.

## 4. Materials and Methods

### 4.1. Animal Collection and Grouping

The Laboratory Animal Management Committee of the Ministry of Health of the People’s Republic of China (SL-2012-42) and the Ethics Committee of Northwestern University have approved the procedures. As previously mentioned by our laboratory [4], 90 ground squirrels were captured in June to July from the Weinan Plain in Shaanxi Province, China. Animals were transported back to the laboratory, weighed, caged, and then provided with water, standard rat food and peanuts. A summer active (SA) group of 30 ground squirrels was held in an animal house, where temperature and light changes due to sunrise and sunset were consistent with local conditions in June/July. From the end of October to the beginning of November, as the remaining 60 ground squirrels became hypothermic, they were transferred to the hibernation room, where temperature was almost the same as the outdoor temperature and the animals were held in totally darkness. The animals entered hibernation, and their body temperatures were monitored by a visual infrared thermometer (Fluke VT04 Visual IR Thermometer, Washington, DC, USA) and recorded at 9:00 a.m. and 9:00 p.m. every day (12 h intervals). These squirrels did not eat or drink after entering torpor and woke up for 12–24 h (interbout arousal) before re-entering torpor. Body weights were measured again before hibernation, and the animals were randomly divided into 3 groups (each group consisted of about 30 ground squirrels): (1) summer active (SA) ground squirrels were sampled from the end of June to the middle of July, when body temperatures were 36–38 °C; (2) the late torpor (LT) group of ground squirrels underwent a torpor–arousal cycle at 5–10 °C for about 60 days, and then went into another torpor bout for 3–4 days with body temperatures of 5–10 °C, when the animals were sampled; (3) the interbout arousal (IBA) squirrels naturally aroused after 60 days of a torpor–arousal cycle, and samples were collected within 12 h at a body temperature of 33–36 °C.

### 4.2. Sample Collection

#### 4.2.1. Carotid Artery Tissue Sample Collection

All squirrels were weighed and anesthetized by intraperitoneal injection dose of 1 mL/100 g of 20% ethyl carbamate. After anesthesia was completed in about 10 min, squirrels were placed on a dissection table and the neck skin was cut for the dissection and isolation of the carotid artery. We placed 0.5 cm carotid artery tissue slices into 2 mL cryovials containing 4% paraformaldehyde and stored them in a refrigerator at 4 °C for subsequent paraffin sectioning and HE staining. The remaining section of each carotid artery (~1 cm) was then placed in a 2 mL cryopreservation tube and snap-frozen in liquid nitrogen, then stored in a −60 °C freezer for subsequent experiments.

#### 4.2.2. Serum Sample Collection

The abdominal cavities of ground squirrels were opened to collect blood from the abdominal aorta and left to stand for 30 min. Samples were then centrifuged at 10,000 rpm at 4 °C for 5 min. The supernatant was removed and centrifuged at 3000 rpm for 5 min at 4 °C. We collected the supernatant from the second centrifugation and distributed it into 200 μL centrifuge tubes, each containing 150 μL, and stored it at −60 °C for subsequent experiments. After all samples were collected, the animals were euthanized by injection of a 20% overdose of ethyl carbamate.

### 4.3. Protein Extraction and Concentration Determination

As mentioned earlier, total soluble protein extracts were prepared from frozen samples of carotid artery from three groups (SA, LT, and IBA) [4]. Frozen tissue samples were ground and lysed in 150 µL RIPA lysis buffer (Heart, Xi’an, China), and 20 µL/mL phosphatase inhibitor and 10 µL/mL protease inhibitor (PMSF) were added and then centrifuged at 15,000 rpm for 15 min at 4 °C followed by collection of the supernatant. The protein concentrations were determined by the BCA method (Boster, Wuhan, China) in an enzyme labeling apparatus. The supernatant was adjusted to the appropriate concentration with 5× SDS and 1× SDS buffer, boiled in a metal bath at 98 °C for 10 min, and then stored at −60 °C for subsequent use.

### 4.4. Western Blots

Proteins were separated using 10% SDS–PAGE gels as described in previous studies [4,5]. Electrophoresis involved 80 V for 30 min, followed by 120 V for 70 min. Then, under the conditions of 20 V, 5 min; 40 V, 20 min; and 60 V, 30 min, the proteins were then transferred onto polyvinylidene difluoride membranes (PVDF membranes 0.20 µm pore size, Merck, Darmstadt, Germany) using a semi-dry transfer apparatus (Bio-Rad, Hercules, CA, USA). Then, we placed the membrane in a solution of 50 mL of 4% PVA-203 (Aladdin, Shanghai, China) of distilled water for 20 min, followed by incubation with rabbit anti-primary antibody diluted in distilled water containing 2% polyvinylpyrrolidone (PVP40, Amresco, Houston, TX, USA) at 4 °C overnight. The primary antibodies were anti-HSP60 (1:2000, Abcam, ab190828, Cambridge, UK) and anti-LONP1 (1:2000, Proteintech, 15440-1-AP, Wuhan, China). Then, we washed the membrane three times with 0.1% TBST on a horizontal oscillator, each time for 10 min, followed by incubation with HRP-conjugated anti-rabbit antibody (1:2000, Zhuangzhi, EK020, Xi’an, China) for 90 min at room temperature, and then washed it three times with 0.1% TBST for ten minutes each time. We used enhanced chemiluminescence reagents (Thermo Fisher Scientific, NCI5079, Waltham, MA, USA) to observe the fluorescence bands and Image Pro Plus software (ipwin32) was used for quantitative analysis. The density of the immunoblot bands in each lane was standardized against the density of the total protein bands in the same lane [4,5].

### 4.5. RNA Extraction and RT-qPCR (Real-Time Fluorescence Quantitative PCR)

#### 4.5.1. RNA Extraction

Total RNA from tissues was extracted using AG RNAex Pro RNA extraction reagent (Accurate Biology, AG21101, Wuhan, China) as described in the literature [88]. Briefly, 60–100 mg of tissue was ground to a powder under liquid nitrogen and then placed into 1.5 mL centrifuge tubes with 1 mL of AG RNAex Pro RNA extraction reagent and left to stand at room temperature for 10 min before being centrifuged for 10 min at 4 °C and 12,000 rpm. Supernatants were then removed and placed into new 1.5 mL centrifuge tubes and then 200 μL of trichloromethane was added to each, vortexed for 30–60 s, left on ice for 10 min, and then centrifuged for 15 min at 4 °C, 12,000 rpm. The supernatant was removed and 500 μL of isopropanol was added, vortexed for 3 s and left on ice for 10 min, then centrifuged for 10 min at 4 °C and 12,000 rpm. The supernatant was discarded, and the precipitate was washed with 75% ethanol and centrifuged for 5 min at 4 °C and 12,000 rpm, which was performed twice. The ethanol was evaporated, and the RNA precipitate was dissolved and diluted with an appropriate amount of DEPC water and then RNA samples were frozen at −60 °C until use.

#### 4.5.2. Reverse Transcription and RT-qPCR

Reverse transcription was performed using the *PerfectStart* Uni RT & qPCR Kit (TransGen Biotech, Beijing, China). The reaction system was as follows: 1 μg of total RNA (the volume addition depended on the concentration of extracted RNA), 4 μL of 5× *TransScript*^®^ Uni All-in-One SuperMix for qPCR, 1 μL of gDNA Remover, and the rest of the 20 μL volume was made up with RNase-free water. Samples were then gently, incubated at 50 °C for 5 min, and then heated at 85 °C for 5 s. After reverse transcription, cDNA was stored at −20 °C.

RT-qPCR was performed using the *PerfectStart* Uni RT & qPCR Kit (TransGen Biotech, Beijing, China). The reaction system was 2× *PerfectStart*^®^ Green qPCR SuperMix 10 μL, upstream and downstream primers (10 μM) of 0.4 μL each, template 1 μL, and nuclease-free water 8.2 μL for a total volume of 20 μL. After addition and centrifugation, eight tubes containing the samples to be tested were placed in the QuantGene 9600 Fluorescent Quantitative Polymerase Reaction (PCR) System and set up for the assay. The relevant procedures and times were as follows: cycling section (initial 94 °C, 30 s, followed by 40 cycles of 94 °C 5 s), then 60 °C 30 s followed by a dissolution section) 95 °C 15 s, 60 °C 1 min, 95 °C 15 s). Fluorescence was selected for SYBR.

### 4.6. Enzyme-Linked Immunosorbent Assay (ELISA)

All inflammatory factors in the carotid artery and melatonin (Fankew, FANKEL, F3532-B, Shanghai, China) in serum were measured using kits (Fankew, Shanghai FANKEL Industrial Co., Ltd., Shanghai, China). Indicators of inflammatory factors in the carotid artery was used to assess IL-1β (F2923-A), IL-6 (F3066-A), IL-10 (F3071-A), CRP (F2957-A), and TNF-α (F3056-A).

### 4.7. Hematoxylin and Eosin (HE) Staining

In brief, as described in the previous literature [4,5], the soaked tissues were removed from paraformaldehyde, trimmed tissues were dehydrated with gradient alcohol, tissues were put into melted and cut paraffin wax and placed in an embedding frame and then into an embedding machine, followed by cooling on a freezer table at −20 °C. After the wax had solidified, wax blocks were removed from the embedding frame and trimmed. The tissue blocks were then placed in a paraffin slicer to cut tissue sections of about 4 μm. The slides were picked up from the slicing machine and placed in an oven at 60 °C to bake the slices and then taken out of the oven and placed at room temperature.

Sections were dewaxed sequentially with the following steps: dewaxing solution I 20 min → dewaxing solution II 20 min → anhydrous ethanol I 5 min → anhydrous ethanol II 5 min → 75% alcohol for 5 min, and a final wash with tap water. Sections were then put into hematoxylin staining solution for 3–5 min, followed by washing with tap water, then differentiated with differentiation solution, washed again with tap water, returned to blue with a re-blue solution, and then rinsed with running water. Tissue sections were then dehydrated by placing them in an 85% to 95% gradient alcohol for 5 min each, then stained in eosin staining solution for 5 min. After staining, we dehydrated the slices and sealed them as follows: anhydrous ethanol I for 5 min → anhydrous ethanol II for 5 min → anhydrous ethanol III for 5 min → dimethyl I for 5 min → xylene II for 5 min for transparency and finally sealed with neutral gum.

Sections were examined microscopically (Nikon Eclipse E100, Nikon, Tokyo, Japan), and images were collected and analyzed (Nikon DS-U3, Nikon, Japan). Finally, the endo-medial thickness was measured using Photoshop software (20.0.0 20180920.r.24 2018/09/20: 1193433 x64) to determine the size of the vascular lumen at different times.

### 4.8. Color Doppler Ultrasonography

Five to seven ground squirrels of varying body weights were selected from each of the three groups, anesthetized using the inhalational anesthetic isoflurane (RWD Life Science Co., Ltd., R510-22, Shenzhen, China), fixed and depilated (Reckitt Benckiser Plc. G20161333, Jinzhou, China). In this experiment, an ultrasound imaging system of VINNO 6LAB (Suzhou, China) model was used, and the 8–18 MHz frequency probe was selected. Before starting the experiment, the number of ground squirrels was registered, the laboratory vascular module was selected, the coupling agent was lightly applied to the probe, and the carotid arteries were probed percutaneously to obtain a clear two-dimensional carotid arterial image. After saving the pictures, the color Doppler was turned on, and the probe position and gain were adjusted slightly to ensure that the blood flow was well filled, and the pulsed spectral Doppler was started to adjust the sampling volume and to ensure that the angle between the direction of the blood flow and the sampling line was not more than 60°. The video and pictures were saved and analyzed to obtain the relevant hemodynamic parameters of the carotid artery in different groups of ground squirrels.

### 4.9. Arterial Blood Gas Measurement

After opening the abdominal cavity of the ground squirrels as described in the previous section, blood was collected from the abdominal aorta using a disposable human Arterial Blood Sampler (*safe* PICO, Radiometer, Copenhagen, Denmark) in accordance with standard procedures. Air from the blood collection tube was quickly removed after collection, then the blood was mixed with a slight shake because it contained gold-plated mixing spheres and electrolyte-balanced dry heparin to reduce the risk of clots in the blood samples. The collected blood sample was placed in the inlet of the ABL9 blood gas analyzer (Radiometer, Denmark), the sample number was entered after automatic aspiration, and the relevant indices were measured.

### 4.10. Cell Culture, Identification, and Sample Collection

After anesthesia, ground squirrel samples from the summer group were soaked in 75% ethanol, as described in the previous section, then the squirrels were placed on a dissection table and the neck skin was cut for carotid artery dissection and isolation. Carotid artery samples were placed in Petri dishes containing DMEM/F12 medium (Pricella, PM150312, Wuhan, China) and rinsed of blood clots inside and outside the vessels. The surrounding connective tissue was stripped and the carotid arteries were transferred to a second Petri dish containing DMEM/F12 medium and the carotid was cleaned again. Artery samples were then transferred to another clean Petri dish and smaller pieces of tissue were cut with ophthalmic scissors. The tissue block was transferred to a 15 mL centrifuge tube containing 3 mL of digestive solution (7.5 mg of type II collagenase) (Solarbio, C8150, Beijing, China) dissolved in 5 mL of DMEM/F12 containing 20% fetal bovine serum (FBS) (Corning, IC-1905, Corning, NY, USA) and digested in an incubator for 6 h. After centrifugation at 1200 rpm for 5 min, the supernatant was removed and 2 mL of DMEM/F12 medium containing 20% FBS was added, gently blowing the dispersed cells to inoculate them into 25 cm^2^ culture flasks, followed by placing them in a 5% CO_2_, 37 °C saturated humidity incubator for cultivation, and they were then passaged when the cells had grown to 80% confluence (7~10 d).

Subsequently, the old medium was aspirated and discarded, and the cells were washed by adding PBS phosphate buffer (Boster, AR0030, Wuhan, China) to adequately wash away residual serum components. Subsequently, 1 mL of 0.25% trypsin (New Cell Molecular Biotech, C100C1, Suzhou, China) was added to the culture flask and digested at 37 °C for 3 min. When the contraction and rounding of the cells was observed under the inverted microscope, the digestion was terminated by adding FBS-containing medium immediately, and the cells were rinsed off from the bottom of the culture flasks, then centrifuged at 1000 rpm for 3 min followed by transfer to new culture flasks at a 1:3 ratio. The solution was changed every 2 days, and the cells were passaged again when they grew to 70% or more confluence. Cell proliferation accelerated on days 3–5, and cell confluence reached more than 80% after 7 days of growth. Experiments were performed with cells from the 3rd to 5th generations.

A cellular immunofluorescence technique was used. Well-grown vascular smooth muscle cells were taken and rinsed with pre-cooled PBS three times for 3 min each time, then 4% paraformaldehyde was used to fix the cells for 30 min. Cells were then rinsed with PBS three times for 3 min each, and the cells were permeabilized with 0.3% Triton X-100 and left to stand for 30 min at room temperature, 500 μL FBS was added at room temperature for 1 h and then discarded, and they were rinsed 3 times for 3 min each with PBS. Smooth muscle cell-specific marker factor anti-a-SMA antibody (1:100, Proteintech, 14395-1-AP, Wuhan, China) was added and incubated at 4 °C overnight, followed by rinsing with PBS 3 times for 3 min each. Secondary antibody (1:100, Zhuangzhi, EK022, Xi’an, China) was then added and incubated for 1 h at room temperature away from light followed by rinsing 3 times with PBS for 3 min each. The samples were sealed with Antifade Mounting Medium with DAPI (Servicebio, G1407, Wuhan, China). Cell morphology and immunohistochemical results were observed under a Nikon ECLIPSE Ts2 microscope (Nikon, Tokyo, Japan).

The cells were randomly divided into four groups: Con, Con + MT, Hypoxia, and Hypoxia + MT. Cells were then cultured for 24 h after passaging, and the control + MT and hypoxia + MT groups were incubated with melatonin (MedChemExpress, HY-B0075, Monmouth Junction, NJ, USA) at a concentration of 1 mmol/L for 24 h. Both hypoxia groups were subjected to hypoxia treatment and cultured in an incubator at 37 °C, 6% O_2_ for 24 h. Cells from the normal group were cultured in an incubator at 5% CO_2_ and 37 °C saturated humidity for 24 h. The well-grown cells were taken, the medium was discarded, and PBS was added and rinsed 2 times, after which the PBS was discarded and the cells were lysed by adding AG RNAex Pro RNA extraction reagent. The solution was later placed into a 1.5 mL centrifuge tube for subsequent RNA extraction experiments.

### 4.11. Statistical Analysis of Data

All statistical testing was performed using SPSS statistical software version 20.0 (IBM, Armonk, NY, USA). Inter group differences were tested using one-way analysis of variance (ANOVA) and Fisher’s least significant difference (LSD). Tamhane was used for testing when homogeneity was not detected. Finally, all data were statistically analyzed and plotted using GraphPad Prism version 8.0 software. Data are presented as mean ± standard deviation (Mean ± SD). *p* < 0.05 was considered a statistically significant difference.

## 5. Conclusions

In summary, our results provide evidence that the intima-media and blood flow velocity of the carotid is significantly reduced, and melatonin accumulates during torpor. In addition, carotid artery PO_2_ and oxygen saturation of the LT group remained close to those in non-hibernators, whereas PO_2_ and oxygen saturation in the SA and IBA groups were lower than in non-hibernators. The carotid artery did not experience oxidative stress or an inflammatory response during the torpor–arousal cycle. Additionally, our study also suggests a possible protective role of melatonin on oxidative stress and inflammation in VSMCs of the carotid artery under hypoxic conditions in vitro. This is the first exploration of the vascular protective function of melatonin in ground squirrels during the torpor–arousal cycle in hibernation. Finally, an understanding the mechanisms by which hibernators maintain carotid structure and function under severe hemodynamic changes during the torpor–arousal cycle may inform strategies for addressing ischemia–reperfusion injury in humans.

## Data Availability

The data presented in this study are available from the corresponding author on request.
